# Effect of Cardiac Rehabilitation Program on Heart Rate Recovery in Coronary Heart Disease 

**Published:** 2015-10-27

**Authors:** Leila Mahdavi Anari, Mahdieh Ghanbari-Firoozabadi, Zahra Ansari, Mahmoud Emami, Mohammadreza Vafaii Nasab, Mahdieh Nemaiande, Fatemeh Boostany, Mohamadreza Neishaboury

**Affiliations:** 1 *Yazd Cardiovascular Research Center, Shahid Sadoughi University of Medical Sciences, Yazd, Iran.*; 2 *School of Medicine, Tehran University of Medical Sciences, Tehran, Iran.*

**Keywords:** *Rehabilitation*, *Heart rate*, *Metabolic syndrome X*

## Abstract

**Background: **It has been suggested that the autonomic system function and the metabolic syndrome can significantly affect patients' survival. The aim of the current study was to investigate the impact of the cardiac rehabilitation program on the autonomic system balance in patients with coronary artery disease.

**Methods:** Patients with a previous diagnosis of coronary artery disease who were referred to the Cardiovascular Rehabilitation Center of Afshar Hospital (Yazd, Iran) between March and November 2011 were enrolled. All the patients participated in rehabilitation sessions 3 times a week for 12 weeks. Heart rate recovery (HRR) was measured as an indicator of the autonomic system balance. In order to calculate HRR, the maximum heart rate during the exercise test was recorded. At the end of the exercise test, the patients were asked to sit down without having a cooldown period and their heart rate was recorded again after 1 minute. The difference between these 2 measurements was considered as HRR.

**Results:** A total of 108 patients, including 86 (79.6%) men and 22 (20.4%) women, completed the rehabilitation course. The mean age of the study participants was 58.25 ± 9.83 years. A statistically significant improvement was observed in HRR (p value = 0.040). Significant declines were also observed in the patients' waist circumference (p value < 0.001) and systolic and diastolic blood pressures (p value = 0.018 and 0.003, respectively). A decreasing trend was observed in the patients' body mass index, but it failed to reach statistical significance (p value = 0.063). No statistically meaningful changes were noted in fasting blood glucose (p value = 0.171), high-density lipoprotein (p value = 0.070), or triglyceride concentrations (p value = 0.149).

**Conclusion:** The cardiac rehabilitation program may help to improve HRR and several components of the metabolic syndrome in patients with coronary heart disease.

## Introduction

Cardiovascular disease (CVD) is one of the most common causes of death throughout the world.^1^^, ^^2^ In 2007, CVD accounted for 33.6% of overall deaths and 15% of health expenditure in the United States.^        3 ^ Coronary heart disease (CHD) is the most common type of CVD.^        1 ^ The exact pathophysiology and genetic causes of CVD remain unclear; however, it has been postulated that the function of the autonomic nervous system has an important role in the development of CVD and affects patients' morbidity and mortality.^   4 ^ Furthermore, the concomitant presence of the components of the metabolic syndrome, namely abdominal obesity, hypertension, impaired glucose metabolism, and insulin resistance, markedly increase the risk.^   5^

The heart rate is predominantly regulated and determined by the autonomic nervous system function. As the heart rate is a significant indicator of myocardial oxygen demand, it is has been demonstrated that individuals with a resting heart rate of more than 90 beats per minute (bpm) have a threefold increased mortality risk compared to those with a rate lower than 60 bpm.^4^^, ^^6^^, ^^7^  Alongside the resting heart rate, heart rate recovery (HRR) defined as the average drop in the heart rate 1 minute after the termination of exercise is an appropriate indicator of the autonomic nervous balance.^8^^, ^^9^ Impaired HRR due to autonomic imbalance is associated with a significant increase in all-cause mortality.^10^^, ^^11^ 

Cardiac rehabilitation is a combination of exercise training and nutritional and psychological counseling aimed at improving the health status of patients with CVD.^   12 ^ The beneficial effects of this technique are proven in patients with a wide range of heart diseases in that this program can result in improved outcome and reduced mortality.^   12^

In the present study, the impact of a supervised cardiac rehabilitation program on the different components of the metabolic syndrome and HRR as an index of the parasympathetic system function was assessed. 

## Methods

All patients with established CHD who were referred to the Cardiovascular Rehabilitation Center of Afshar Hospital (Yazd, Iran) between March and November 2011 were included. The patients had previously received appropriate treatment and been candidated for participation in the rehabilitation program based on the decision of the attending cardiologists. Of note, all the patients included in the present study had previously undergone coronary revascularization via either coronary artery bypass graft (CABG) or percutaneous transluminal coronary angioplasty (PTCA). The rehabilitation program was initiated 2 weeks after PTCA and 8 weeks after CABG according to the recommendations of The American Association of Cardiovascular and Pulmonary Rehabilitation (AACVPR) (http://www.aacvpr.org). The participants were asked regarding previous diagnoses of diabetes, hypertension, and hyperlipidemia and smoking habits. Patients with a history of diagnosed lung, liver, or kidney disease as well as those who refused to participate in the study were excluded. Patients who did not complete the course of rehabilitation were not included in the final analysis. The benefits of the cardiac rehabilitation program were explained to the study participants, and informed consent was obtained prior to enrollment. The study protocol was approved by the Ethics Committee of Shahid Sadoughi University of Medical Sciences. 

All the patients underwent transthoracic echocardiography, and the left ventricular ejection fraction was measured by using the biplane Simpson method. The patients' weight and height were measured using a digital scale and a stadiometer, respectively. The body mass index (BMI) was calculated as weight in kilograms divided by height squared in meters. The waist circumference was measured with an inflexible measurement tape mid-way between the iliac crest and the lower costal margin. The blood pressure was measured using a standard mercury sphygmomanometer 3 times at 5-minute intervals after 10 minutes of rest. The mean values of the second and third measurements were considered as the mean systolic and diastolic blood pressure. The levels of triglycerides (TG), high-density lipoprotein (HDL), low-density lipoprotein (LDL), and total cholesterol were determined using enzymatic methods (Pars Azmun commercial kits, Karaj, Iran). The glucose oxidase test was employed to measure fasting blood glucose (FBG) concentrations. All the assessments, with the exception of height, were repeated at the end of the trial. The criteria of The International Diabetes Federation (IDF) were used to define the metabolic syndrome.^   13 ^ The patients were considered as having the metabolic syndrome if they had central obesity (defined as waist circumference ≥ 94 cm in men and 80 ≥ cm in women) and any 2 of the following 4 conditions: 1) TG level ≥ 150 mg/dl, 2) systolic blood pressure ≥ 130 mmHg or diastolic blood pressure ≥ 85 mmHg or previously diagnosed hypertension, 3) FBG ≥ 100 mg/dl or previously diagnosed diabetes, and 4) HDL < 40 mg/dl in males and < 50 mg/dl in females.

All the patients were asked to participate in the hospital-based exercise training program 3 times a week for 12 weeks. The exercise sessions were initiated with 5 minutes of warm-up exercise such as walking and stretching, followed by treadmill walking or cycling supervised by a nurse and a physiotherapist. The intensity of the exercises was monitored in terms of the participants’ pulse rate, electrocardiogram, and symptoms. The duration of each session increased from 20 minutes in the first week to 60 minutes in the last week. Each session was ended with 5 minutes of cooldown exercise. The patients were visited by a nutritionist and received a dietary recommendation based on their BMI, waist circumference, and laboratory assessments in order to modify their dietary habits.

The study participants were also provided with comprehensive recommendations for diet and exercise by a single specialist. The recommendations were focused on the following key objectives: 1) gradual weight loss (about 7% of body weight), 2) adoption of a balanced diet (emphasizing on reducing the consumption of simple carbohydrates and saturated fatty acids along with an increasing intake of whole grains and dietary fibers), and 3) following a regular exercise plan of moderate intensity (50%–70% of maximum heart rate), at least 30 minutes daily, 5 times a week. Moreover, resistance training was recommended if there were no contraindications.

In order to calculate HRR, the maximum heart rate during the exercise test was recorded. At the end of the exercise test, the patients were asked to sit down without having a cooldown period, and the heart rate was recorded again after 1 minute. The difference between these 2 measurements was considered as HRR. The patients were then advised to do 5 minutes of cooldown exercise. 

The statistical analyses were carried out using Statistical Package for the Social Sciences (SPSS) for Windows, version 20.0 (IBM Inc., New York, US). The continuous variables are reported as mean and standard error of mean, while the categorical variables are presented as percentage. The paired *t*-test and the McNemar test were employed to assess the efficacy of exercise training on the studied variables. The impact of previous treatment was assessed through the categorization of the patients into 2 groups (CABG and PTCA); and via the analysis of covariance, the final HRR was compared between the 2 mentioned groups, while adjusting for the baseline values. A p value < 0.05 was considered statistically significant in all the analyses. 

## Results

A total of 257 patients with CAD were referred to our cardiac rehabilitation center in Yazd, Iran, during the study period. Among those, 108 patients who met the study criteria were recruited and participated in exercise training 3 times a week, for 12 weeks (36 sessions). The mean age of the study participants was 58.25 ± 9.83 years. The study population was comprised of 86 (79.6%) men and 22 (20.4%) women. Concerning the 2 main types of previous surgical interventions, 78 (72.2%) patients underwent CABG and 30 (27.8%) PTCA. The demographic information of the patients is presented in Table 1.

**Table 1:** Baseline characteristics of the study participants (n=108)*

Age (mean±SD)	58.25±9.83
Gender	
Male	86 (79.6)
Female	22 (20.4)
Type of previous treatment	
CABG	78 (72.2)
PTCA	30 (27.8)
Medications	
Beta-blocker	85 (78.7)
CCB	14 (13.0)
ACEI	13 (12.0)
ARB	42 (38.9)
Diuretic	28 (25.9)
Statin	92 (85.2)
Diabetes	41 (38.0)
Hypertension	60 (55.5)
Hyperlipidemia	36 (33.3)
Smoking	29 (26.9)
Metabolic syndrome	85 (78.7)
Family history of CVD	36 (33.3)

CABG, Coronary artery bypass grafting; PTCA, Percutaneous transluminal coronary angioplasty; CCB, Calcium channel blocker; ACEI, Angiotensin-converting enzyme inhibitor; ARB, Angiotensin receptor blocker; CVD, Cardiovascular disease

* Data are presented as mean±SD or n (%)

The changes in the different components of the metabolic syndrome during the study course are depicted in Table 2. Eighty-five (78.7%) patients had the metabolic syndrome at the beginning of the study according to the IDF guideline. The rehabilitation program resulted in a significant decline in 3 out of the 7 components (waist circumference and resting systolic and diastolic blood pressures). Moreover, a prominent decrease was observed in the waist circumference of the patients (p value < 0.001). Although a declining trend was observed in the BMI and also an increasing trend in HDL concentrations, the differences failed to reach statistical significance (p value = 0.063 and 0.070, respectively). The number of patients with the metabolic syndrome declined to 80 (74.0%) at the end of the study, but the reducing trend did not show a significant change (p value = 0.063).

Prior to rehabilitation initiation, left ventricular ejection fraction was 48.44% ± 1.01 and increased to 49.50% ± 0.91 after the last session (p value = 0.158). The measurement of HRR indicated an improvement in the autonomic system function in that it increased from 13.76 ± 1.38 bpm to 17.07 ± 1.33 bpm after session 36. The statistical analyses revealed a meaningful change in HRR (p value = 0.040) (Table 2). As was mentioned previously, the patients were categorized based on the types of previous major treatment protocol (CABG and PTCA). The improvement in HRR was compared between these 2 groups, and no statistical differences were observed (p value = 0.338).

**Table 2:** Assessing the effect of rehabilitation program on study outcomes during the study course*

	Week 1	Week 12	P Value
Heart function			
LVEF (%)	48.44±1.01	49.50±0.91	0.158
Autonomic system index			
HRR (%)	13.76±1.38	17.07±1.33	0.040
Components of the metabolic syndrome			
BMI (Kg/m^2^)	28.12±0.40	27.93±0.41	0.063
Waist (cm)	99.04±1.01	97.30±1.00	< 0.001
FBG (mg/dl)	114.90±3.51	110.04±3.24	0.171
TG (mg/dl)	169.33±9.62	161.05±7.57	0.149
HDL (mg/dl)	40.29±0.97	41.88±1.02	0.070
Systolic blood pressure (mmHg)	156.19±2.12	150.56±2.06	0.018
Diastolic blood pressure (mmHg)	84.72±0.80	82.05±0.75	0.003

LVEF; Left ventricular ejection fraction, HRR, Heart rate recovery; BMI, Body mass index; FBG, Fasting blood glucose; TG, Triglyceride; HDL, High-density lipoprotein

* Data are presented as mean±standard error of mean

## Discussion

The current study aimed to investigate the effect of a supervised exercise training program on the autonomic system function and the components of the metabolic syndrome. Patients with established CHD who had received appropriate treatment based on the decision of their physician were enrolled. HRR was measured in all the patients in the first and last sessions of the exercise program as an indicator of autonomic balance. Significant changes were observed in the patients' HRR values, regardless of considering HRR as a discrete or a continuous variable. There was no change in the drug prescription of the study participants in order to minimize the confounding effect of medication on the study results. On the other hand, there were changes made in the dietary habits of the study population by a nutritionist as a routine and recommended part of the rehabilitation program and such changes do not have the mentioned effect.^   12 ^ Additionally, the patients experienced a significant decline in their waist circumference and systolic and diastolic blood pressures. 

During exercise, the heart rate is increased due to the suppression of the parasympathetic tone and the stimulation of the sympathetic system, while after the termination of exercise, the autonomic changes that happen during exercise are reversed and this helps decrease the heart rate.^   9 ^ Accordingly, HRR is a simple indicator of autonomic regulation and balance. Previous studies have shown that HRR ≤ 12 bpm in the first several minutes after exercise termination is associated with a higher mortality risk. Chen et al.^   14 ^ assessed the impact of HRR on patients' long-term mortality between 2 groups of patients with myocardial ischemia: One group underwent early revascularization, while the other patients who did not receive any invasive treatment were considered as the non-revascularization group. The authors concluded that the first group had a better outcome; nevertheless, a decreased HRR was associated with higher mortality and it diminished the difference in the mortality rate between the 2 groups. In healthy individuals and athletes, the heart rate drops rapidly after exercise, showing the capacity of their cardiac and nervous systems to maintain balance.^   15^

Previous studies have implicated that the rehabilitation program can enhance HRR and, thus, improve patients' outcome. Ribeiro et al.,^16^ conducting a clinical trial comprised of 38 patients after their first myocardial infarction in order to assess the effect of cardiac rehabilitation on the autonomic function, observed that 8 weeks of exercise training resulted in a significant decline in the systolic blood pressure and increase in HRR. Their results are in good agreement with ours. Streuber et al.^17^ noted the same effect in 45 patients with heart failure after 12 weeks of rehabilitation. Myers et al.^   18 ^ measured HRR 6 minutes after the termination of the exercise session at intervals of 1 minute in 24 patients and observed that 12 patients who participated in the rehabilitation program had a significantly faster decline in the heart rate 2 to 6 minutes after the end of exercise compared to the control group. However, the authors noted no difference after 1 minute between the 2 groups.

In one study which assessed the prevalence of the metabolic syndrome in patients with acute coronary events, almost 67% of the 277 patients involved had the metabolic syndrome, which was inversely related to age. Exercise training and lifestyle change conferred improvement in several components of the metabolic syndrome.^19^ Several authors have reported the same effect after exercise training.^20^^-^^22^ It has been proven that the metabolic syndrome can negatively affect the prognosis of patients with CHD. Nigam et al.^23^ reported that in 24958 patients diagnosed with CHD, the metabolic syndrome was associated with a higher all-cause mortality rate over a follow-up duration of 12.6 years. Our results are in good agreement with previous studies inasmuch as there were significant declines in the waist circumference and the systolic and diastolic pressures. Additionally, cardiac rehabilitation reduced the BMI levels and HDL concentrations, although the difference did not reach statistical significance.

The major limitation of the current study is the absence of a control group. Further studies that recruit a group of patients with known CAD who do not participate in hospital-based cardiac rehabilitation are warranted. Only through comparing the results between those who do and those who do not participate in a supervised exercise training program will we be able to evaluate more accurately the impact of hospital-based rehabilitation and its beneficial effects on autonomic systems. 

## Conclusion

Our results demonstrated that a supervised cardiac rehabilitation program improved the autonomic function as measured by HRR. Additionally, significant improvements were observed in several components of the metabolic syndrome. 

## Notes:

***This paper should be cited as:*** Mahdavi Anari L, Ghanbari-Firoozabadi M, Ansari Z, Emami M, Vafaii Nasab MR, Nemaiande M, Boostany F, Neishaboury MR, Ebrahim B. Effect of Cardiac Rehabilitation Program on Heart Rate Recovery in Coronary Heart Disease. J Teh Univ Heart Ctr 2015;10(4):176 -181.
